# Hierarchical analysis of genetic structure in the habitat-specialist Eastern Sand Darter (*Ammocrypta pellucida*)

**DOI:** 10.1002/ece3.1392

**Published:** 2015-01-13

**Authors:** Robert Ginson, Ryan P Walter, Nicholas E Mandrak, Courtney L Beneteau, Daniel D Heath

**Affiliations:** 1 Great Lakes Institute for Environmental Research, University of Windsor401 Sunset Avenue, Windsor, Ontario, N9B 3P4, Canada; 2 Great Lakes Laboratory for Fisheries and Aquatic Sciences, Fisheries and Oceans Canada867 Lakeshore Road, Burlington, Ontario, L7R 4A6, Canada; 3 Department of Biological Sciences, University of Windsor401 Sunset Avenue, Windsor, Ontario, N9B 3P4, Canada

**Keywords:** Eastern Sand Darter, fragmented habitat, genetic diversity, genetic structure, stratified dispersal

## Abstract

Quantifying spatial genetic structure can reveal the relative influences of contemporary and historic factors underlying localized and regional patterns of genetic diversity and gene flow – important considerations for the development of effective conservation efforts. Using 10 polymorphic microsatellite loci, we characterize genetic variation among populations across the range of the Eastern Sand Darter *(Ammocrypta pellucida*), a small riverine percid that is highly dependent on sandy substrate microhabitats. We tested for fine scale, regional, and historic patterns of genetic structure. As expected, significant differentiation was detected among rivers within drainages and among drainages. At finer scales, an unexpected lack of within-river genetic structure among fragmented sandy microhabitats suggests that stratified dispersal resulting from unstable sand bar habitat degradation (natural and anthropogenic) may preclude substantial genetic differentiation within rivers. Among-drainage genetic structure indicates that postglacial (14 kya) drainage connectivity continues to influence contemporary genetic structure among Eastern Sand Darter populations in southern Ontario. These results provide an unexpected contrast to other benthic riverine fish in the Great Lakes drainage and suggest that habitat-specific fishes, such as the Eastern Sand Darter, can evolve dispersal strategies that overcome fragmented and temporally unstable habitats.

## Introduction

Specialized microhabitat dependence presents a formidable challenge to species conservation in changing environments. For some species, the coupling of microhabitat specialization with increased habitat degradation and fragmentation can initiate or accelerate declines in population size and, ultimately, local extirpation. Microhabitat specialization provides an extreme example of local adaptation and raises questions about mechanisms that allow the persistence of such specialized life histories in variable environments. Characterization of genetic structure and gene flow among fragmented habitats can yield important information for the conservation of such microhabitat-dependent species. Specifically, a hierarchical analysis can reveal the relative importance of large-scale historical processes (e.g., climatic, hydrological, geographic) and more contemporary fine-scale processes (e.g., in-stream barriers) in shaping overall patterns of genetic variation (Wiens 1997; Monaghan et al. 2002).

Molecular genetic methods can provide nonlethal means to successfully characterize many aspects of ecosystem processes and population connectivity for species at risk, including landscape effects on genetic substructure (Cook et al. 2007; Caldera and Bolnick 2008), historical influences on contemporary population structure (Poissant et al. 2005; Stepien et al. 2007; Boizard et al. 2009), colonization patterns and alternative dispersal pathways (Mäkinen et al. 2006), and species introductions (Dlugosch and Parker 2008; Beneteau et al. 2012). Quantifying range-wide population connectivity provides valuable information on species dynamics and aids in the identification of isolated populations requiring special conservation attention (Manel et al. 2003; Cook et al. 2007; Storfer et al. 2007). Most importantly, genetic identification of fine-scale dispersal and gene flow patterns among fragmented populations may indicate natural or assisted recolonization potential for extirpated habitat patches (Bohonak 1999; Palsbøll et al. 2007).

Connectivity among populations depends on species-specific dispersal capabilities (Watanabe et al. 2010) and barriers to dispersal, which may disrupt gene flow by limiting among-population movements (McGlashan and Hughes 2001; Poissant et al. 2005; Johansson et al. 2008). Populations in freshwater ecosystems often show low levels of connectivity and high levels of genetic divergence as these ecosystems commonly rely on linear corridors of stream connectivity (Ward et al. 1994). The array of connectivity pathways among freshwater habitats ranging from small streams to lakes provides a variety of potential dispersal barriers for aquatic organisms (Caldera and Bolnick 2008). For habitat-specific fishes, such as darters, the loss or degradation of specialized habitats may disrupt not only within-river genetic connectivity but also natural metapopulation dynamics (Turner and Trexler 1998).

The Eastern Sand Darter (*Ammocrypta pellucida*) is a small benthic riverine fish federally listed as threatened in Canada and listed as threatened in many states in its American distribution (Grandmaison et al. 2004; Committee on the Status of Endangered Wildlife in Canada (COSEWIC) 2011). *A. pellucida* exhibits a unique burying behavior associated with sandy substrates, which may limit its potential for passive drift dispersal (Daniels 1989), but does exhibit a nonbenthic larval period (Simon and Wallus 2006). Tagging studies on *A. pellucida* showed no evidence for adult movement among sand bars during the summer months (Finch 2009). Those findings in conjunction with the patchy distribution of riverine sand bar habitat are expected to promote genetic divergence among adult assemblages. However, early life-stage dispersal and/or mixing of separate sand bar populations during the winter months has been suggested, but not tested, and both possibilities could facilitate mixing among sand bar populations (Simon and Wallus 2006). At a larger scale, the species range encompasses a patchy network of inhabited and uninhabited rivers, with the loss of suitable habitat largely attributed to anthropogenic pressures (Grandmaison et al. 2004; COSEWIC 2009). In the last century, *A. pellucida* has experienced a nearly 40% reduction in distribution, including extirpation from three Canadian river systems: Catfish Creek, Big Otter Creek (Lake Erie drainage), and the Ausable River (Lake St. Clair drainage).

Here, we assess the degree of population divergence for *A. pellucida* across its natural range. Using data from 10 microsatellite loci from individuals sampled from 39 sites, we aim to (1) characterize contemporary population connectivity through analyses of genetic structure and dispersal and (2) determine the relative influence of historic (postglaciation) colonization patterns versus current connectivity processes on drainage-level genetic structure. In general, we expect high genetic structure among sand bar sites for *A. pellucida*, even at small spatial scales, due to the species’ restricted distribution to sandy substrate habitats. Additionally, we expect high levels of genetic divergence among regions as a result of population isolation and decline (Grandmaison et al. 2004; Committee on the Status of Endangered Wildlife in Canada (COSEWIC) 2011), although postglacial recolonization from different refugia and from different patterns of historic connectivity can also affect present-day genetic structure in *A. pellucida*. Consequently, the combination of habitat specialization and fragmentation within rivers, historic genetic patterns of connectivity and declining population sizes in most rivers reinforces the conservation and evolutionary importance of characterizing genetic structure among these populations.

## Methods

### Sampling

**Figure 1:**
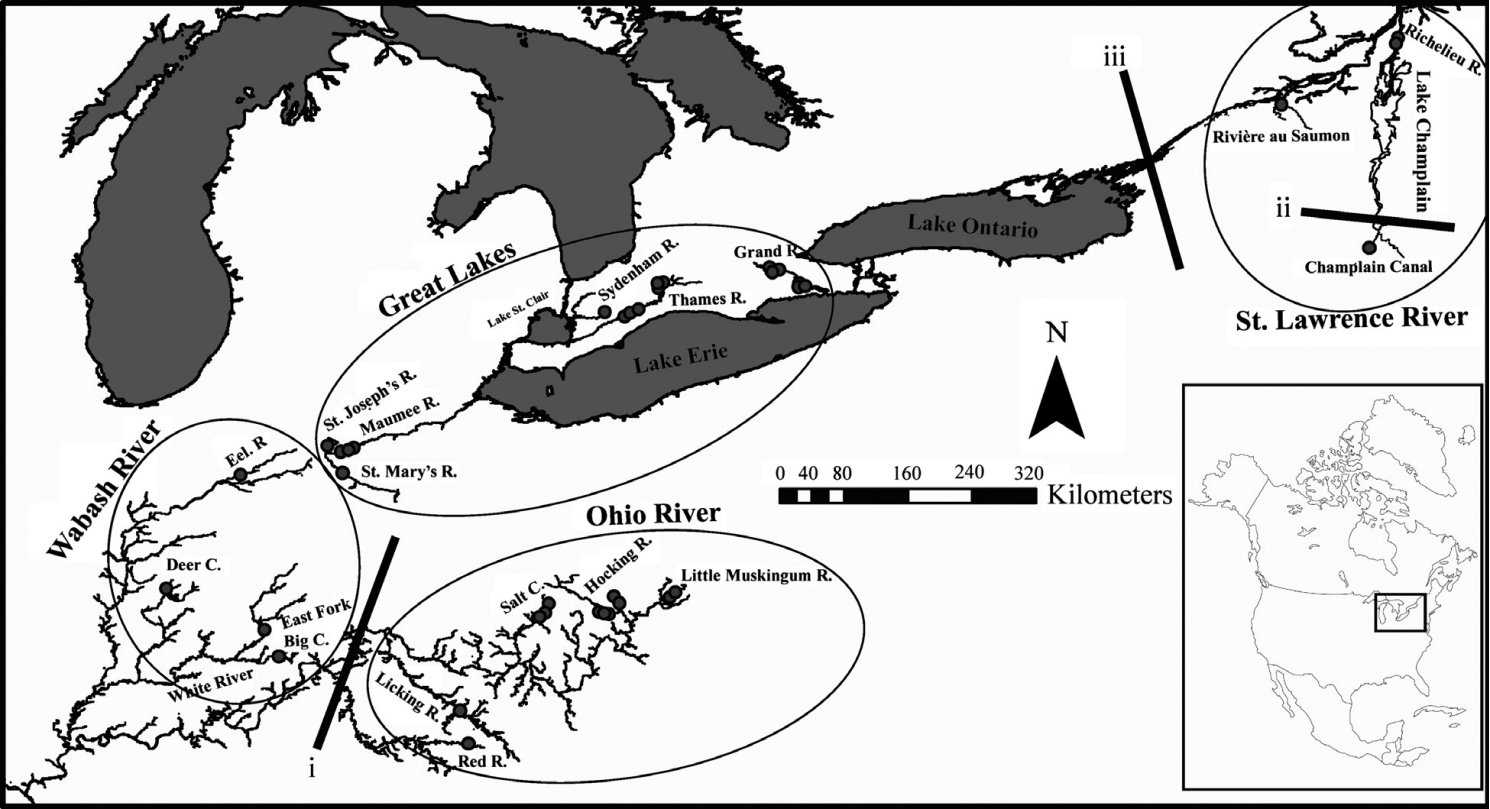
Eastern Sand Darter collection sites (filled dots) across the species range in North America. Ellipses identify the four sampled drainages: Great Lakes drainage (Lake Erie/Lake St. Clair), Ohio drainage, Wabash drainage, and St. Lawrence drainage (St. Lawrence River/Lake Champlain). Three major genetic discontinuities identified using BARRIER software are shown as black solid lines on the map.

Sampling efforts focused on rivers recently reported to harbor *A. pellucida* populations (Grandmaison et al. 2004; Fisheries and Oceans Canada 2012), with targeted sampling directed to sand bars at depositional river bends. Hierarchical sampling definitions used in this study include sample sites (e.g., HR1), within rivers (e.g., Hocking River), within drainages (e.g., Ohio drainage). Sampling occurred in four drainages across the species range (Fig.1): (1) Ohio drainage (Little Muskingum River, Hocking River, Salt Creek, Red River, Licking River); (2) Wabash drainage (Eel River, East Fork White River, Deer Creek, Big Creek); (3) Great Lakes drainage (Maumee River, Grand River, Thames River, Sydenham River); and (4) St. Lawrence drainage (Richelieu River, Rivère au Saumon, Champlain Canal). Ohio and Wabash drainages were categorized as separate drainages because the sampled rivers within those drainages are separated by over 1000 km. Fish were caught with a bag seine net (dimensions: wings 15 × 3 m with 0.64 cm mesh and 1.5 × 1.5 × 1.5 m bag with 0.32 cm mesh) or using a Missouri trawl specialized for benthic fish collection. Upon collection, a small pelvic fin clip was taken from each fish and preserved in 95% ethanol for subsequent DNA analysis. After a short recovery period in freshwater tanks, fish were then returned to their original habitats.

### DNA extraction and genotyping

Fish were genotyped at 10 microsatellite loci, five of which were developed specifically for *A. pellucida* (Esd3, Esd13, Esd17, Esd18, Esd25) and an additional five loci from other darter species (Esc132b, EosC6, EosC112, EosD107, EosD11; see Table S1). Microsatellite loci discovery and primer development included the extraction of DNA followed by enrichment for repeat sequences using a protocol adapted from Fischer and Bachman (1998). Briefly, genomic DNA was digested with RsaI and ligated to MluI adapter–primer complexes (5′-CTCTTGCTTACGCGTGGACTA-3, 5′-pTAGTCCACGCGTAAGCAAGAGCACA-3′). DNA fragments were hybridized with 5′-biotinylated oligo (GACA_4_) probes, captured with streptavidin-coated beads (Roche, Indianapolis, USA), and enriched using polymerase chain reactions (PCR). The resulting enriched DNA library was inserted into TOPO vectors and transformed into One Shot competent *Escherichia coli* cells (Invitrogen, Burlington, ON, Canada). Clone inserts were amplified using M13 universal forward and reverse primers and sequenced at Genome Quebec Innovation Centre (McGill University, Montreal, Canada). Microsatellite primer pairs were designed and optimized for polymorphism and ease of PCR amplification. PCR amplification of all ten microsatellite loci used in this study was performed in 12.75 *μ*L reactions containing approximately 50–100 ng template DNA, 0.25 *μ*L of 0.5 *μ*mol/L dye-labeled forward primer, 0.25 *μ*L of 0.5 *μ*mol/L reverse primer, 200 *μ*mol/L of each dNTP, various concentrations of MgCl_2_ (Table S1), and 0.25U Taq DNA polymerase (Applied Biosystems, Foster City, CA) in a 1× PCR buffer. PCR thermal cycler profiles consisted of an initial denaturing period at 94°C for 120 sec followed by 35 cycles of 94°C for 30 sec, various annealing temperatures for each primer (Table S1) for 45 sec, 30 sec at 72°C, and 90 sec at 72°C at the final extension period. Dye-labeled PCR products were visualized on a LiCor 4300 DNA analyzer (LiCor Biosciences, Inc. Lincoln, Nebraska, USA). Individual genotypes were determined by scoring allele sizes using GENE IMAGIR 4.05 software (Scanalytics Inc. Fairfax, VA, USA).

### Marker validation

Genotype data for each site were tested for the presence of null alleles, allele scoring error, and large allele dropout using MICROCHECKER 2.2.3 (Van Oosterhout et al. 2004). All pairs of microsatellite loci were analyzed for linkage disequilibrium in ARLEQUIN 3.01 (Excoffier et al. 2005). Departures from Hardy–Weinberg equilibrium (HWE) were assessed for all possible locus-by-site combinations using the Markov chain Monte Carlo (MCMC) method (100,000 dememorization steps; 1,000,000 Markov chain steps) in ARLEQUIN. HWE departure significance and all subsequent pairwise comparisons were adjusted for multiple simultaneous tests using sequential Bonferroni corrections (Rice 1989).

### Genetic analyses

#### Genetic differentiation

Genetic differentiation was estimated by calculating pairwise *F*_ST_ values (Weir and Cockerham 1984) among all sites in ARLEQUIN. To quantify genetic differentiation among rivers, sites within each river were combined and mean pairwise *F*_ST_ estimates were calculated among rivers using ARLEQUIN. Global *F*_ST_ values were calculated among all rivers within each of the four drainages (to compare levels of divergence among rivers within drainages), with significance determined by jackknifing across all loci at the 95% confidence interval in FSTAT (Goudet 2001). Allelic richness (*A*_R_), number of alleles (*A*), observed heterozygosity (*H*_O_), expected heterozygosity (*H*_E_), inbreeding coefficient (*F*_IS_) were also calculated in FSTAT.

#### Isolation by distance

Rivers with more than three sampling sites were tested for adherence to an isolation by distance (IBD) model of migration–drift equilibrium. IBD was determined using the association between linearized genetic differentiation (*F*_ST_/(1 − *F*_ST_); Slatkin 1995) and hydrological distances (km) among sites and the shortest hydrological distances between sites, with a Mantel test for significance (9999 permutations) in GENALEX 6.0 (Peakall and Smouse 2006). However, *A. pellucida* prefers shallow, sandy habitats so hydrological distances were determined at the drainage scale using two methods: shallow water restriction (assumes individuals avoid open water and calculates shoreline distances through lakes) and open-water dispersal (uses the shortest water distances among rivers including dispersal through open water).

#### Hierarchical genetic analysis

Analysis of molecular variance (AMOVA) was used to hierarchically partition genetic variation within each drainage into three levels: among rivers, among sites within rivers, and within sites using ARLEQUIN. We also identified the number of population genetic clusters using the Bayesian-based clustering program STRUCTURE 2.3.4 (Pritchard et al. 2000). When the model of *K* = 1 could be rejected, we used the Δ*K* method to select *K* (Evanno et al. 2005) as implemented in STRUCTURE HARVESTER (Earl and von Holdt 2012), and the process was repeated on all recovered *K*s in a hierarchical approach as described in Roy et al. (2012) – see Fig. S1. STRUCTURE runs were performed in five iterations for each *K*, each with a 100,000 burn-in, 1,000,000 Markov chain Monte Carlo (MCMC) generations, allele frequencies correlated, and admixture allowed. The number of genetic clusters was allowed to range from *K* = 1 (range-wide panmixia) to the total number of rivers plus one (*K* = 17). Runs were compiled using full searches in CLUMPP1.1.2 (Jakobsson and Rosenberg 2007) and plotted with DISTRUCT1.1 (Rosenberg 2004). To explore within-river structure, we performed additional full dataset STRUCTURE runs using the site of capture as a location prior. We also performed STRUCTURE runs on smaller river-specific datasets to further resolve within-river structure. To visualize the relative divergence of the sites and rivers, we performed a principal coordinate analysis (PCoA) using a pairwise matrix of F_ST_ values in GENALEX. We used BARRIER 2.2 (Manni et al. 2004) and the landscape genetic approach of Monmonier's maximum difference algorithm across the range to identify breaks in gene flow patterns among geographically close sites. In BARRIER, pairwise estimates of *F*_ST_ were mapped onto a matrix of the population geographic coordinates (latitude and longitude), and the Monmonier's maximum difference algorithm identified which of the borders between neighboring populations exhibited anomalous genetic divergence relative to spatial separation.

#### Contemporary versus historic influences

As the genetic signature from historic colonization patterns may persist and affect estimation of contemporary connectivity patterns, population genetic structure should be analyzed to test for possible large-scale patterns consistent with historic gene flow patterns (Duvernell et al. 2008). To determine the potential influence of historic drainage connectivity on contemporary genetic structure, we tested the relative partitioning of genetic variance identified by historic versus contemporary groups of sites using AMOVA. The contemporary site grouping (based on present-day drainage connectivity) has three groups: (1) sites in the Great Lakes drainage; (2) sites in the Ohio and Wabash drainages; and (3) sites in the St. Lawrence drainage. Under the historic connectivity hypothesis, drainages were grouped based on preglaciated patterns of water drainage (Underhill 1986; Mandrak and Crossman 1992): (1) Great Lakes and Wabash drainage sites; (2) Ohio drainage sites; and (3) St. Lawrence drainage sites. The proportion and significance of genetic variance partitioned within and among groups for each hypothesized grouping pattern was assessed hierarchically using AMOVA in ARLEQUIN. If the historic group model explains more variance than the contemporary river connectivity model, then historic effects still influence the structure of genetic connectivity across the range. We used the corrected Akaike information criterion (AIC_c_; Burnham and Anderson 1998) to identify the best-fit model based on variance explained (Halverson et al. 2007).

#### Dispersal

To quantify patterns of dispersal among the sites, rivers, and drainages, we performed a self-exclusion analysis in GENECLASS. Individual fish were excluded/assigned to sites, rivers, and drainages using the Bayesian method of Rannala and Mountain (1997) and the Paetkau et al. (2004) Monte Carlo simulation as implemented in GENECLASS 2 (Piry et al. 2004) with *α *= 0.05, using 100,000 simulated individuals. A fish was considered excluded from a site, river, or drainage of capture if the Bayesian probability was less than 0.05, and assigned if the Bayesian probability was equal to or greater than 0.05: This results in conservative exclusion outcomes.

## Results

### Sampling and marker assessment

A total of 1051 individuals were collected from 39 sites in 16 rivers across the entire species range from June 2010 to November 2011 (Fig.1). Across sites, microsatellite allelic richness ranged from 2.64 to 5.87 (Table1). Significant departures from HWE were found in eight of 390 possible locus-by-site combinations following Bonferroni correction (*P* < 0.001; Table S1). Five sites were monomorphic at locus Esd3 (HRc1, HRc2, HRm3, HRm1, LK), while site CC was monomorphic at locus EosC6. Seven of the locus-by-site deviations from HWE were attributed to null alleles by MICROCHECKER; however, no single locus had more than two sites deviating from HWE, and we conclude that null alleles are not substantially influencing our results. Significant (*P* < 0.001) linkage disequilibrium was determined for five of 390 possible locus-by-locus combinations over all the sites, with no two loci identified in linkage disequilibrium for more than one site indicating that our marker loci likely are not linked.

**Table 1:** Descriptions of 39 Eastern Sand Darter collection sites sampled in this study (see Fig.1 for geographic locations). Drainage refers to groups of rivers described in the text. For each river sampled, a description of the capture sites is given (site IDs, GPS coordinate, number of individuals (*N*), corrected allelic richness (*A*_R_), number of alleles (*A*), observed heterozygosity (*H*_O_), expected heterozygosity (*H*_E_), inbreeding coefficient (*F*_IS_), bold type indicates significant values (*P* < 0.05)

Drainage	River name	Site ID	Latitude	Longitude	*N*	*A*_R_	*A*	*H*_O_	*H*_E_	*F*_IS_
Wabash	Eel river	ER	40°49′41″	−86°06′50″	30	4.71	68	0.676	0.683	0.007
	East Fork White R.	EF	39°08′19″	−85°53′38″	32	5.53	91	0.694	0.747	0.073
	Big Creek	BC	38°48′33″	−85°38′38″	39	5.87	108	0.728	0.741	0.014
	Deer Creek	DC	39°30′02″	−86°55′49″	32	5.84	99	0.712	0.727	0.017
Ohio	Red river	Rd	37°49′11″	−83°34′33″	17	5.31	69	0.714	0.777	**0.120**
	Licking river	Lk	38°12′30″	−83°40′49″	19	5.33	74	0.580	0.687	0.010
	Salt Creek	SC1	39°26′00″	−82°40′48″	16	5.42	72	0.704	0.700	−0.030
		SC2	39°20′59″	−82°40′40″	30	5.26	85	0.657	0.683	0.010
		SC3	39°19′50″	−82°40′56″	20	5.74	87	0.670	0.716	0.066
	Hocking river	HRm1	39°18′03″	−81°57′50″	25	5.26	88	0.624	0.636	0.019
		HRm2	39°17′44″	−81°56′14″	36	5.28	93	0.597	0.652	0.064
		HRm3	39°17′48″	−81°54′05″	38	5.41	101	0.602	0.636	0.050
		HRc1	39°19′49″	−81°53′19″	37	5.67	113	0.664	0.662	−0.018
		HRc2	39°19′22″	−81°53′06″	28	5.50	96	0.640	0.654	−0.001
	Little Muskingum R.	LM1	39°24′42″	−81°21′31″	17	5.55	75	0.769	0.719	−0.116
		LM2	39°24′25″	−81°21′26″	38	5.63	101	0.683	0.677	−0.017
		LM3	39°24′14″	−81°21′27″	24	5.78	93	0.676	0.688	−0.019
Great Lakes	Maumee river	SM	40°53′41″	−85°00′26″	31	4.76	69	0.635	0.667	0.045
		SJ	41°06′44″	−85°07′05″	35	5.05	77	0.654	0.710	0.077
		MA1	41°05′03″	−85°01′11″	35	4.91	73	0.670	0.700	0.036
		MA2	41°06′34″	−84°57′47″	32	4.92	76	0.675	0.691	0.013
		MA3	41°07′50″	−84°56′06″	28	4.94	71	0.708	0.702	−0.010
	Sydenham river	Syd	42°38′49″	−82°00′35″	12	5.47	68	0.600	0.702	0.135
	Thames river	THu1	42°55′55″	−81°25′35″	28	5.78	103	0.661	0.721	**0.085**
		THu2	42°55′24″	−81°25′53″	27	5.58	93	0.640	0.708	**0.094**
		THu3	42°54′30″	−81°25′30″	30	5.45	98	0.679	0.704	0.031
		THd1	42°39′38″	−81°42′28″	32	5.60	99	0.741	0.727	−0.045
		THd2	42°38′33″	−81°42′15″	24	5.30	84	0.730	0.712	−0.070
		THd3	42°39′39″	−81°44′17″	21	5.66	88	0.757	0.736	−0.060
	Grand river	GRu1	43°07′40″	−80°11′57″	25	5.56	88	0.731	0.738	−0.011
		GRu2	43°06′02″	−80°14′26″	17	5.26	77	0.694	0.726	0.045
		GRu3	43°05′47″	−80°12′59″	27	5.49	88	0.740	0.747	−0.008
		GRd1	42°59′04″	−79°52′25″	29	5.52	95	0.749	0.749	0.008
		GRd2	42°58′15″	−79°52′48″	29	5.51	96	0.741	0.742	0.001
		GRd3	42°57′31″	−79°52′12″	22	5.62	89	0.695	0.752	0.065
St. Lawrence	Rivière au Saumon	RAS	44°59′57″	−74°30′38″	21	4.26	61	0.631	0.621	−0.032
	Richelieu river	RR1	45°38′06″	−73°11′26″	30	4.61	76	0.658	0.627	−0.062
		RR2	45°39′13″	−73°12′01″	27	3.94	62	0.560	0.570	0.003
	Champlain canal	CC	43°21′09″	−73°29′44″	11	2.64	29	0.491	0.445	−0.108

### Genetic structure

#### Genetic differentiation

Within-river pairwise *F*_ST_ values among sites were generally low and nonsignificant (after Bonferroni correction), although some (<10%) between-site *F*_ST_ values were substantial and significant (Table2). The pairwise exact tests of allele frequency distribution differences resulted in a higher proportion of significant between-site differences (51% significant; Table2); this is likely due to the much higher sensitivity of the exact test. Pairwise *F*_ST_ values among rivers within each drainage were substantially higher (0.021–0.18; Table3) and all but three pairwise *F*_ST_ values were highly statistically significant after Bonferroni correction (88%; Table3). *F*_ST_ values were even higher when rivers were compared among drainages (Table3). Global *F*_ST_ values across all rivers within each drainage show that the St. Lawrence drainage region had the highest overall genetic differentiation (*F*_ST_ = 0.11 ± 0.022) compared to the other drainages (Great Lakes drainage *F*_ST_ = 0.049 ± 0.011; Ohio drainage *F*_ST_ = 0.054 ± 0.011; Wabash drainage *F*_ST_ = 0.044 ± 0.014). This pattern persisted even when geographic distances were corrected to 1000 km (St. Lawrence drainage *F*_ST_ = 0.44; Great Lakes drainage *F*_ST_ = 0.099; Ohio drainage *F*_ST_ = 0.090; Wabash drainage *F*_ST_ = 0.069).

**Table 2:** Within-river genetic differentiation among Eastern Sand Darter sample sites from three different drainages (Ohio, Great Lakes, and St. Lawrence; note the Wabash drainage is not shown as each river had only one sampled site). Within each river, pairwise *F*_ST_ values (below diagonal) were calculated among sites. Significant results for pairwise *F*_ST_ estimates were also calculated, and significant results (after Bonferroni correction) are indicated in boldface type

Drainage							
		LM1	LM2	LM3			
Ohio drainage	LM1	–					
	LM2	0.007	–				
	LM3	0.003	–0.002	–			
		HRc1	HRc2	HRm1	HRm2	HRm3	
	HRc1	–					
	HRc2	0.009	–				
	HRm1	–0.003	** 0.020**	–			
	HRm2	0.005	** 0.021**	0.001	–		
	HRm3	0.003	** 0.015**	–0.002	0.001	–	
		SC1	SC2	SC3			
	SC1	–					
	SC2	0.005	–	N			
	SC3	0.003	–0.003	–			
		THu1	THu2	THu3	THd1	THd2	THd3
Great Lakes	THu1	–					
	THu2	0.004	–				
	THu3	0.003	0.015	–			
	THd1	0.003	0.005	0.002	–		
	THd2	0.001	0.009	0.000	0.000	–	
	THd3	0.008	0.006	–0.003	–0.002	–0.007	–
		GRu1	GRu2	GRu3	GRL1	GRL2	GRL3
	GRu1	–					
	GRu2	–0.006	–				
	GRu3	0.005	–0.005	–			
	GRL1	0.009	–0.002	–0.002	–		
	GRL2	0.004	–0.005	–0.004	0.001	–	
	GRL3	0.005	–0.002	–0.008	0.002	–0.005	–
		SJ	MA1	MA2	MA3	SM	
	SJ	–					
	MA1	0.001	–				
	MA2	0.001	–0.001	–			
	MA3	0.007	0.000	0.012	–		
	SM	0.012	0.009	** 0.014**	** 0.024**	–	
		RR1	RR2				
St. Lawrence R.	RR1	–					
	RR2	0.005	–				

Bold indicates significance following Bonferroni correction (*P* < 0.01, 0.005, 0.01, 0.003, 0.003, 0.005, 0.05) below diagonal.

Bold indicates significant pairwise exact test (*P* < 0.05) above diagonal.

**Table 3:** Pairwise *F*_ST_ values calculated among all sampled rivers (16 rivers with sample sites combined, drainages are indicated) for Eastern Sand Darter. Bold-face type indicates significance following Bonferroni correction (*P* < 0.001)

	Wabash				Ohio					Great Lakes				St. Lawrence		
	ER	EF	BC	DC	Lk	Rd	LM	HR	SC	MA	Syd	TH	GR	RAS	RR	CC
ER	**–**															
EF	**0.075**	**–**														
BC	**0.085**	0.011	**–**													
DC	**0.076**	**0.024**	0.009	**–**												
Lk	**0.160**	**0.103**	**0.081**	**0.078**	**–**											
Rd	**0.144**	**0.089**	**0.069**	**0.063**	**0.032**	**–**										
LM	**0.103**	**0.072**	**0.063**	**0.042**	**0.075**	**0.049**	**–**									
HR	**0.164**	**0.119**	**0.085**	**0.073**	**0.080**	**0.046**	**0.053**	**–**								
SC	**0.153**	**0.139**	**0.123**	**0.112**	**0.069**	**0.060**	**0.075**	**0.081**	**–**							
MA	**0.081**	**0.047**	**0.058**	**0.077**	**0.148**	**0.145**	**0.120**	**0.165**	**0.162**	**–**						
Syd	**0.062**	**0.071**	**0.084**	**0.084**	**0.172**	**0.159**	**0.121**	**0.175**	**0.154**	**0.054**	**–**					
TH	**0.053**	**0.047**	**0.054**	**0.053**	**0.123**	**0.110**	**0.083**	**0.126**	**0.134**	**0.050**	0.021	**–**				
GR	**0.099**	**0.077**	**0.090**	**0.088**	**0.156**	**0.149**	**0.109**	**0.168**	**0.165**	**0.090**	**0.044**	**0.055**	**–**			
RAS	**0.114**	**0.070**	**0.056**	**0.060**	**0.159**	**0.147**	**0.115**	**0.130**	**0.171**	**0.096**	**0.116**	**0.081**	**0.105**	**–**		
RR	**0.148**	**0.096**	**0.098**	**0.086**	**0.184**	**0.170**	**0.118**	**0.146**	**0.190**	**0.125**	**0.143**	**0.098**	**0.093**	**0.060**	**–**	
CC	**0.259**	**0.170**	**0.184**	**0.193**	**0.279**	**0.267**	**0.224**	**0.237**	**0.281**	**0.243**	**0.289**	**0.204**	**0.205**	**0.155**	**0.175**	**–**

#### Isolation by distance

**Figure 2:**
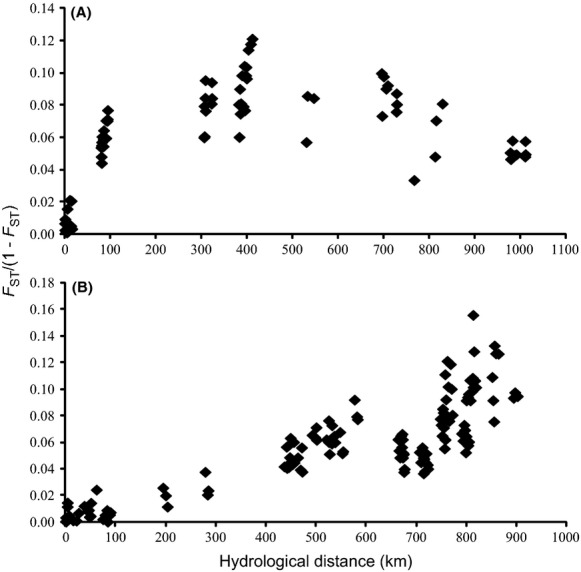
Isolation by distance (IBD) relationships for Eastern Sand Darter sampled from the (A) Ohio drainage (*P* < 0.004) and (B) Great Lakes drainage (shallow water distance, *P* < 0.0001).

Due to limited numbers of within-river sample sites, IBD was only assessed in three rivers (Maumee, Grand and Thames rivers) in the Great Lakes drainage and one river (Hocking River) in the Ohio drainage. Significant within-river IBD was found for the Maumee River (*R*^2^ = 0.61, *P* = 0.039); however, no significant IBD was found in the Hocking River. Low *F*_ST_ values among all sites in the Thames and Grand rivers resulted in a lack of IBD correlation for both rivers (*R*^2^ = 0.035, *P* = 0.21 and *R*^2^ = 0.021, *P* = 0.21, respectively). Mantel tests of IBD among rivers within each drainage showed that both the Ohio drainage (*R*^2^ = 0.18, *P* = 0.004) and Great Lakes drainage (*R*^2^ = 0.80, *P* = 0.0001, straight line and *R*^2^ = 0.79, *P* = 0.0001, shallow water distances) had significant IBD (Fig.2). Neither the Wabash drainage (*R*^2^ = 0.79, *P* = 0.125) nor St. Lawrence drainage (*R*^2^ = 0.52, *P* = 0.084) adhered to an IBD pattern of divergence.

#### Range-wide genetic structure

**Figure 3:**
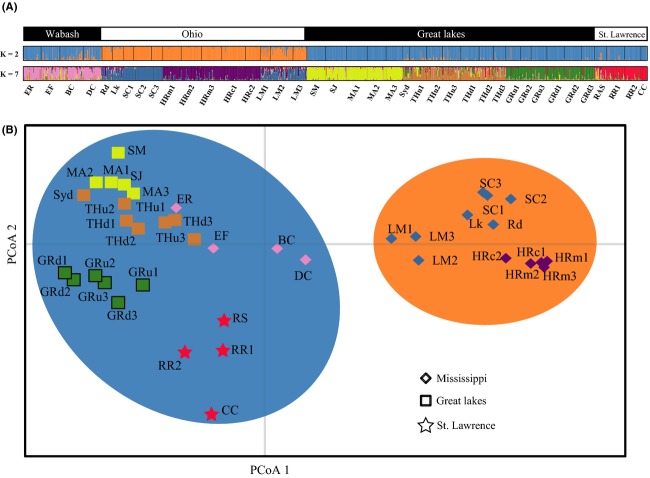
Range-wide genetic structure analysis of the Eastern Sand Darter; Panel (A): results of STRUCTURE analysis using 39 sample sites from 16 rivers across species range. STRUCTURE simulation summary for each sample site, with different colors showing each genetic cluster at *K* = 2 and *K* = 7, respectively. Panel (B): principle coordinate analysis (PCoA) performed using pairwise *F*_ST_ among all sampled sites across the species range. The range was separated into three drainages: St. Lawrence, Great Lakes, and Mississippi (combination of Wabash and Ohio drainages). The proportion of genetic variance explained by the first two axes is 62.7%.

Individual AMOVAs for each drainage revealed low among-site (within river) genetic variance: Ohio drainage (0.42%, *P* = 0.002), Great Lakes drainage (0.31%, *P* = 0.008), St. Lawrence drainage (0.46%, *P* = 0.132). However, the among-river genetic variance component was 15–20 times higher in the three drainages: Ohio drainage (6.50%, *P* < 0.0001), Great Lakes drainage (6.29%, *P* < 0.0001), St. Lawrence drainage (10.52%, *P* < 0.0001). The highest proportion of genetic variance in all analyses was attributed to the within-sites component: Ohio drainage (93.09, *P* < 0.0001), Great Lakes drainage (93.39%, *P* < 0.0001), St. Lawrence drainage (89.02%, *P* = 0.116). The Wabash drainage was excluded from the AMOVA analysis because the within-river sample sites were not replicated (Table1). STRUCTURE revealed two cluster patterns with approximately equal likelihood (based on Δ*K*; Fig. S1). At *K* = 2, STRUCTURE grouped sites from the Wabash drainage with the Great Lakes and St. Lawrence drainages, while the Ohio drainage sites grouped separately (Fig.3A). Our hierarchical analysis also recovered seven clusters (*K* = 7) with population delineation at chiefly the river level (Fig.3A). STRUCTURE runs using location priors did not produce differing results. STRUCTURE runs on reduced datasets from individual systems also supports genetic structure at the river level. Principal coordinate analysis (PCoA) revealed a similar delineation among sites in the Ohio drainage versus the remaining range-wide sites along the first axis (Fig.3B). The PCoA also showed a clear division of the St. Lawrence drainage from other sites (Fig.3B). PCoA further supported the STRUCTURE results, as the Wabash sites clustered with the Great Lakes sites (Fig.3B). BARRIER identified three major genetic breaks: The first separated the Ohio drainage from the rest of the range, and the second genetic barrier isolated the Champlain Canal site from all other sites (Fig.1). The third genetic barrier isolated the St. Lawrence drainage from the Great Lakes drainage (Fig.1).

#### Contemporary versus historic influences

AMOVA results for both historic and contemporary connectivity models yielded highly significant models; however, a greater proportion of the among-group genetic variance was explained when the groups reflected the historic connection between the Wabash River and Great Lakes drainages (8.15%, *P* < 0.0001), as opposed to contemporary connectivity alone (5.09%, *P* < 0.0001). The ΔAIC_c_ between the two AMOVAs was 12.6, highly supportive for the historic model (AIC_c_ = 2763) versus the contemporary model (AIC_c_ = 2686) of genetic variation. Both AMOVA analyses revealed substantial and very similar components of the genetic variance attributed to within-river variation (historic = 87%, *P* < 0.0001 and contemporary = 88%, *P* < 0.001).

#### Dispersal

**Figure 4:**
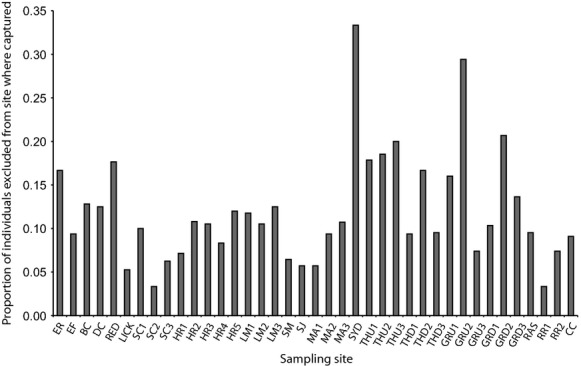
GENECLASS self-exclusion analysis including Eastern Sand Darters from 16 rivers (39 sampled sites) across their distribution showing the proportion of individuals sampled from each site whose Bayesian probability of self-assignment is less than 0.05, that is, they are likely strays.

GENECLASS assignment resulted in a total of 120 individuals conservatively excluded from their “site of capture”, ranging from 3.0% to 33% of the individuals caught at a given site (Fig4). Of the fish excluded from their site of capture, most were assigned to another site within the same river they were captured from, or to an adjacent river (Table2). A total of 20 fish failed to assign to any site sampled within the study, of those most were captured in the Grand River (Tables2 and 4).

**Table 4:** Summary of GENECLASS exclusion/assignment results for all hierarchically sampled Eastern Sand Darters. Individuals were considered excluded from “site of capture” if their Bayesian probability was less than 0.05, those excluded individuals were then assigned to another site(s) if *P* > 0.05 for a given site. A total of 20 individuals could not be assigned to any site; therefore, their origin is unknown

	Excluded	Source of excluded fish			
River	Total	Within river	Adjacent river	Multiple origins	Unknown
SC	3	1	0	1	1
HR	18	2	5	8	3
LM	9	8	0	1	0
MA	8	3	0	3	2
ThR	25	10	11	0	4
GR	21	9	4	1	7
RR	3	0	0	0	3

## Discussion

Freshwater fish species inhabiting formerly glaciated regions commonly exhibit genetic signatures that reflect the influence of historical glacial refugia and recolonization patterns (Costello et al. 2003; Poissant et al. 2005; Stepien et al. 2007; Boizard et al. 2009; Shikano et al. 2010; Walter et al. 2012). Our data reveal the persisting influence of historic, postglacial drainage patterns on large-scale (range wide) patterns of genetic divergence. On the other hand, our analyses show little or no evidence for contemporary connectivity (i.e., gene flow) among drainages perhaps, not surprisingly, given the large hydrological distances between most rivers in the study, the limited dispersal capabilities of this small benthic fish, and unsuitable habitat separating some of the drainages. However, our data show extensive genetic connectivity among habitat patches within all sampled rivers, regardless of anthropogenic barriers (e.g., low Grand River genetic differentiation despite separation of sites by a dam). The high dispersal among sand bars identified in our analysis challenges previous conclusions regarding the sedentary nature of *A. pellucida*. In general, the nature of the freshwater “landscapes” promotes low genetic structure within rivers and higher genetic structure among rivers in freshwater fishes (Mäkinen et al. 2006; Cook et al. 2007; Shikano et al. 2010). Published exceptions to this pattern, where sculpins and darters exhibit high within-river genetic structure, are attributed to anthropogenic barriers to dispersal (Hänfling and Weetman 2006; Beneteau et al. 2006). For *A. pellucida*, we did not expect low levels of within-river genetic structure as their suitable habitat is fragmented within rivers (both naturally and anthropogenically). However, our analyses clearly indicate substantial movement of individuals among the sampled habitats. The lack of genetic structure within the sampled rivers likely reflects species-specific dispersal that counteracts patchy habitat distribution.

For *A. pellucida*, a combination of long and short within-river dispersal (or “stratified dispersal”) may contribute to the lack of within-river genetic structure and this, in turn, would act to buffer individual sand bar populations from genetic drift effects and loss of genetic diversity (Bronnenhuber et al. 2011). Hydrological distances among sites within rivers were generally not positively correlated with genetic differentiation. This lack of IBD is consistent with stratified within-river dispersal, restricting genetic differentiation among sample sites, a pattern that is apparent in the Thames and Grand rivers. Generally speaking, within-river IBD is expected unless dispersal distances are larger than the spatial extent of the study area or if sufficient long-distance dispersal events occur to swamp genetic drift effects (McGlashan and Hughes 2001). Disruptions to within-river IBD could also result from recurring population bottlenecks, preventing migration–drift equilibrium, as suggested for other darter species (Turner and Trexler 1998; Johnson et al. 2006). However, we observed no evidence for low genetic diversity or elevated F_IS_ values (see Table1). Thus, we conclude that the lack of within-river genetic structure in populations likely reflects primarily stratified dispersal. Within-river movements of adults may be directly influenced by their dependence on a temporally unstable habitat (depositional sand bars). That is, fish are forced to disperse when their preferred habitat is locally lost or degraded, a plausible scenario for sand deposition-based habitat. Within-river movement would be further promoted by a largely nonbenthic larval stage where downstream larval drift could facilitate gene flow within rivers (Simon and Wallus 2006). Independent of the mechanism behind within-river dispersal, the high genetic connectivity demonstrated here indicates that reintroduction efforts using fish taken from the same river would hold little genetic risk as the fish are already well mixed and might be better characterized as assisted dispersal rather than reintroduction.

At the drainage scale, among-river IBD patterns in the Ohio and Great Lakes drainages suggest that hydrological distances restrict genetic connectivity among rivers and that the river populations appear to be at, or near, dispersal–drift equilibrium. Very few among-river migrants were identified range wide, indicating that dispersal among rivers is infrequent and results in little among-river gene flow. No difference in the IBD relationship was observed for shallow water versus straight-line dispersal pathways in the Great Lakes drainage (the only drainage with a large lake in this study), suggesting that open-lake habitat does not represent a major barrier to the dispersal–drift equilibrium for *A. pellucida*. Based on the pattern of among-river genetic divergence, reintroduction plans should give preference for populations as geographically close to the reintroduction site as possible, assuming fish from the same river cannot be used.

As expected, among-river genetic structure was substantial and significant in all sampled drainages, similar to other darter species within the Great Lakes drainage (Greenside Darter, Beneteau et al. 2009; Rainbow Darter, *Etheostoma caeruleum*, Haponski et al. 2009). A variety of biotic and abiotic habitat characteristics likely restrict the ability of darters to disperse through freshwater drainages (Jackson et al. 2001), including fast river flow and unsuitable habitat (Cook et al. 2007; Zamudio et al. 2009). We found only three exceptions to substantial among-river genetic divergence in *A. pellucida*: The first occurred between the Thames and Sydenham rivers, and the other two were among rivers in the Wabash drainage. Low genetic differentiation between the Thames and Sydenham river populations can be explained by either dispersal between the spatially close river mouths in the shallow Lake St. Clair or headwater connections from natural floods or anthropogenic fish movement. The genetic similarity between these two rivers most likely reflects a headwater connection or human-mediated transfer, as suggested for Greenside Darter (Beneteau et al. 2009). The relatively high genetic connectivity among rivers in the Wabash drainage may result from few anthropogenic barriers (e.g., dams, weirs), lower stream flow rates and shorter hydrological distances separating rivers. Unfortunately, our data do not allow us to conclusively identify the source of the anomalous genetic connectivity among rivers.

The genetic divergence of *A. pellucida* in the Ohio River drainage from the remainder of the species range is likely a result of long-term isolation. Much of the Ohio River drainage, including the sites in our study, were never glaciated, whereas the remaining sites in our study (i.e., Wabash, Great Lakes, and St. Lawrence drainages) were covered most recently by the Wisconsinan continental ice sheet (Trautman 1981, Burr and Page 1986). Following the Wisconsinan glacial retreat (approximately 14,000 years ago), *A. pellucida* would have colonized the Wabash and Great Lakes/St. Lawrence drainages from the Mississippian refugium (Underhill 1986; Mandrak and Crossman 1992) The genetic similarity between sites in the Wabash and Great Lakes drainages likely reflects the historical connection of the Great Lakes and Wabash drainages following the end of the Wisconsinan glacial period, when excess water from the glacial Lake Maumee (ancestor of present-day Lake Erie) drained into what is now the Wabash River (Underhill 1986). This historic connection between the Wabash and Great Lakes drainages has been previously hypothesized to be a major connection for aquatic organisms recolonizing the Great Lakes from the Mississippian refugium (Underhill 1986; Mandrak and Crossman 1992) and to have driven genetic similarities between mussel populations in the Wabash and Great Lakes drainages (Graf 2002; Elderkin et al. 2007). Another important genetic influence of glacial colonization pathways on populations involves isolated, or “disjunct”, species range patterns (Witt et al. 2011). A major genetic break identified in this study occurred between the St. Lawrence drainage and the remainder of the species range. *A. pellucida* are thought to have colonized Lake Champlain and the St. Lawrence River from the Mississippian glacial refugium through either the Mohawk River of the glacial Lake Iroquois (present-day Lake Ontario), 12,000–13,500 years ago, and subsequently through Lampsilis Lake (present-day St. Lawrence River), 8500–10,000 years ago (Underhill 1986). Alternatively, *A. pellucida* in the St. Lawrence drainage may have derived from an Atlantic Coastal refugium (Committee on the Status of Endangered Wildlife in Canada (COSEWIC) 2011). The reduced genetic diversity exhibited by the St. Lawrence drainage populations, coupled with their genetic divergence and low connectivity, indicate that these populations merit increased conservation attention. The genetic divergence of the Quebec ESD also supports the recent identification of two conservation units in Canada (termed “designatable units”), the Quebec and Ontario population have separate status and recovery plans (COSEWIC 2009).

Our study emphasizes the blending of contemporary and historic influences on the genetic structure of *A. pellucida* populations throughout the species range. Based on the pattern of among-river genetic divergence, supplementation and reintroduction plans for extirpated systems with currently suitable habitat (Dextrase et al. 2014) should give preference for not only geographically proximal populations, but also those with contemporary and historical genetic connections. This study highlights the influence of historic drainage connectivity and not only reveals genetic cohesiveness between previously connected drainages (e.g., the Wabash–Maumee historical connection) but also provides insight into the negative genetic effects of range isolation in disjunct drainages (e.g., St. Lawrence drainage). Small-scale analyses showed an unexpected lack of genetic structure at the within-river level, consistent with substantial and ongoing dispersal and hence connectivity. The within-river dispersal likely results from the temporal instability of specialized habitat (sand bars) possibly combined with larval drift. Our hierarchical range-wide analysis of the genetic structure in a habitat-specific species clearly demonstrates that species-specific life-history traits, such as dependence on specific habitats, can strongly affect genetic diversity patterns, particularly when the preferred habitat is fragmented and temporally unstable.

## Data Accessibility

Microsatellite genotype data available on Dryad: DOI 10.5061/dryad.q2d3v.

## Conflict of Interest

None declared.

## Supporting Information

**Figure S1.** Hierarchical analysis of population genetic structure using STRUCTURE and STRUCTURE Harvester. Recovered K's were separately analyzed until K = 1 could not be rejected.

**Table S1.** Characterization of ten microsatellite markers used for genetic analysis of *Ammocrypta pellucida*.

## References

[b100] Beneteau CL, Mandrak NE, Heath DD (2009). The effects of river barriers and range expansion of the population genetic structure and stability in Greenside Darter (*Etheostoma blennioides*) populations. Cons. Gen.

[b1] Beneteau CL, Walter RP, Mandrak NE, Heath DD (2012). Range expansion by invasion: genetic characterization of invasion of the greenside darter (*Etheostoma blennioides*) at the northern edge of its distribution. Biol. Invasions.

[b2] Bohonak AJ (1999). Dispersal, gene flow, and population structure. Q. Rev. Biol.

[b3] Boizard J, Magnan P, Angers B (2009). Effects of dynamic landscape elements on fish dispersal: the example of creek chub (*Semotilus atromaculatus*. Mol. Ecol.

[b4] Bronnenhuber JE, Dufour BA, Higgs DM, Heath DD (2011). Dispersal strategies, secondary range expansion and invasion genetics of the nonindigenous round goby, *Neogobius melanostomus*, in Great Lakes tributaries. Mol. Ecol.

[b5] Burnham KD, Anderson DR (1998). Model selection and inference: a practical information-theoretic approach.

[b6] Burr BM, Hocutt CH, Wiley OH, Page LM (1986). Zoogeography of fishes of the lower Ohio River-Upper Mississippi basin. The zoogeography of North American freshwater fishes.

[b7] Caldera EJ, Bolnick DI (2008). Effects of colonization history and landscape structure on genetic variation within and among threespine stickleback (*Gasterosteus aculeatus*) populations in a single watershed. Evol. Ecol. Res.

[b101] COSEWIC (2009). http://www.sararegistry.gc.ca.

[b8] Committee on the Status of Endangered Wildlife in Canada (COSEWIC) (2011). http://www.sararegistry.gc.ca.

[b9] Cook BD, Bunn SE, Hughes JM (2007). Molecular genetic and stable isotope signatures reveal complementary patterns of population connectivity in the regionally vulnerable southern pygmy perch (*Nannoperca australis*. Biol. Conserv.

[b10] Costello AB, Down TE, Pollard SM, Pacas CJ, Taylor EB (2003). The influence of history and contemporary stream hydrology on the evolution of genetic diversity within species: an examination of microsatellite DNA variation in bull trout, *Salvelinus confluentus* (Pisces: Salmonidae). Evolution.

[b11] Daniels RA (1989). Significance of burying in *Ammocrypta pellucida*. Copeia.

[b12] Dextrase AJ, Mandrak NE, Schaefer JA (2014). Modelling occupancy of an imperilled stream fish at multiple scales while accounting for imperfect detection: implications for conservation. Freshw. Biol.

[b13] Dlugosch KM, Parker IM (2008). Founding events in species invasions: genetic variation, adaptive evolution, and the role of multiple introductions. Mol. Ecol.

[b14] Duvernell DD, Lindmeier JB, Faust KE, Whitehead A (2008). Relative influences of historical and contemporary forces shaping the distribution of genetic variation in the Atlantic killifish, *Fundulus heteroclitus*. Mol. Ecol.

[b15] Earl DA, von Holdt BM (2012). STRUCTURE HARVESTER: a website and program for visualizing STRUCTURE output and implementing the Evanno method. Conserv. Genet. Resour.

[b16] Elderkin CL, Christian AD, Vaughn CC, Metcalfe-Smith JL, Berg DJ (2007). Population genetic of the freshwater mussel, *Amblema plicata* (Say 1817) (Bivalvia: Unionidae): evidence of high dispersal and post-glacial colonization. Conserv. Genet.

[b17] Evanno G, Regnaut S, Goudet J (2005). Detecting the number of clusters of individuals using the software STRUCTURE: a simulation study. Mol. Ecol.

[b18] Excoffier L, Laval G, Schneider S (2005). Arlequin (version 3.0): an integrated software package for population genetics analysis. Evol. Bioinf. Online.

[b19] Finch MR (2009). University of Waterloo.

[b20] Fischer D, Bachman K (1998). Microsatellite enrichment in organisms with large genomes (*Alliumcepa L.)*. Biotechniques.

[b21] Fisheries and Oceans Canada (2012). Recovery strategy for the eastern sand darter (Ammocrypta pellucida) in Canada: Ontario populations.

[b23] Goudet J (2001). http://www.unil.ch/popgen/softwares/fstat.html.

[b24] Graf DL (2002). Occasional Papers on Mollusks, The Department of Mollusks, Museum of Comparative Zoology, Harvard University, Cambridge, MA.

[b25] Grandmaison D, Mayasich J, Etnier D (2004).

[b26] Halverson K, Heard SB, Hason JD, Stireman JO (2007). Origins, distribution, and local co-occurrence of polyploid cytotypes in *Solidago altissima* (Asteraceae). Am. J. Bot.

[b27] Hänfling B, Weetman D (2006). Concordant genetic estimators of migration reveal anthropogenically enhanced source-sink population structure in the river sculpin, *Cottus gobio*. Genetics.

[b28] Haponski AE, Bollin TL, Jedlicka MA, Stepien CA (2009). Landscape genetic patterns of the rainbow darter *Etheostoma caeruleum*: a catchment analysis of mitochondrial DNA sequences and nuclear microsatellites. J. Fish Biol.

[b29] Jackson DA, Peres-Neto PR, Olden JD (2001). What controls who is where in freshwater fish communities – the roles of biotic, abiotic, and spatial factors. Can. J. Fish. Aquat. Sci.

[b30] Jakobsson M, Rosenberg NA (2007). *CLUMPP*: a cluster matching and permutation program for dealing with label switching and multimodality in analysis of population structure. Bioinformatics.

[b31] Johansson ML, Banks MA, Glunt KD, Hassel-Finnegan HM, Buonaccorsi VP (2008). Influence of habitat discontinuity, geographical distance, and oceanography on fine-scale population genetic structure of copper rockfish (*Sebastes caurinus*. Mol. Ecol.

[b32] Johnson RL, Mitchell RM, Harp GL (2006). Genetic variation and genetic structuring of a numerically declining species of darter, *Etheostoma moorei* Raney & Suttkus, endemic to the Upper Little Red River, Arkansas. Am. Midl. Nat.

[b33] Mäkinen HS, Cano JM, Merilä J (2006). Genetic relationships among marine and freshwater populations of the European three-spined stickleback *(Gasterosteus aculeatus)* revealed by microsatellites. Mol. Ecol.

[b34] Mandrak NE, Crossman EJ (1992). Postglacial dispersal of freshwater fishes in Ontario. Can. J. Zool.

[b35] Manel S, Schwartz MK, Luikart G, Taberlet P (2003). Landscape genetics: combining landscape ecology and population genetics. Trends Ecol. Evol.

[b36] Manni F, Guérard E, Heyer E (2004). Geographic patterns of (genetic, morphologic, linguistic) variation: how barriers can be detected by using Monmonier's algorithm. Hum. Biol.

[b37] McGlashan DJ, Hughes JM (2001). Low levels of genetic differentiation among populations of the freshwater fish *Hypseleotris compressa* (Gobiidae: Eleotridinae): implications for its biology, population connectivity and history. J. Hered.

[b39] Monaghan MT, Spaak P, Robinson CT, Ward JV (2002). Population genetic structure of 3 alpine stream insects: influences of gene flow, demographics, and habitat fragmentation. J. N. Am. Benthol. Soc.

[b40] Paetkau D, Slade R, Burdens M, Estoup A (2004). Genetic assignment methods for the direct, real time estimation of migration rate: a simulation-based exploration of accuracy and power. Mol. Ecol.

[b41] Palsbøll PJ, Bérubé M, Allendorf FW (2007). Identification of management units using population genetic data. Trends Ecol. Evol.

[b42] Peakall R, Smouse PE (2006). GENALEX 6: genetic analysis in Excel. Population genetic software for teaching and research. Mol. Ecol. Notes.

[b43] Piry S, Alapetite A, Cornuet JM, Paetkau D, Baudouin L, Estoup A (2004). GENECLASS 2: a software for genetic assignment and first generation migrant detection. J. Hered.

[b44] Poissant J, Knight TW, Ferguson MM (2005). Nonequilibrium conditions following landscape rearrangement: the relative contribution of past and current hydrological landscapes on the genetic structure of a stream-dwelling fish. Mol. Ecol.

[b45] Pritchard JK, Stephens M, Donnelly P (2000). Inference of population structure using multilocus genotype data. Genetics.

[b46] Rannala B, Mountain JL (1997). Detecting immigration by using multilocus genotypes. Proc. Natl Acad. Sci. USA.

[b48] Rice WR (1989). Analyzing tables of statistical tests. Evolution.

[b49] Rosenberg NA (2004). Distruct: a program for the graphical display of population structure. Mol. Ecol. Notes.

[b50] Roy D, Hurbut TR, Ruzzante DE (2012). Biocomplexity in a demersal exploited fish, white hake (*Urophycis tenuis*): depth-related structure and inadequacy of current management approaches. Can. J. Fish. Aquat. Sci.

[b51] Shikano T, Shimada Y, Herczeg G, Merila J (2010). History vs. habitat type: explaining the genetic structure of European nine-spined stickleback (*Pungitius pungitius)* populations. Mol. Ecol.

[b52] Simon TP, Wallus R (2006). Reproductive biology and early life history of fishes in the Ohio River drainage- Percidae- perch, pikeperch and darters.

[b53] Slatkin M (1995). A measure of population subdivision based on microsatellite allele frequencies. Genetics.

[b54] Stepien CA, Murphy DJ, Strange RM (2007). Broad- to fine-scale population genetic patterning in the smallmouth bass *Micropterus dolomieu* across the Laurentian Great Lakes and beyond: an interplay of behaviour and geography. Mol. Ecol.

[b55] Storfer A, Murphy MA, Evans JS, Goldberg CS, Robinson S, Spear SF (2007). Putting the “landscape” in landscape genetics. Heredity.

[b102] Trautman MB (1981). The fishes of Ohio.

[b57] Turner TF, Trexler JC (1998). Ecological and historical associations of gene flow in darters (Teleostei: Percidae). Evolution.

[b58] Underhill JC, Hocutt CH, Wiley EO (1986). The fish fauna of the Laurentian Great Lakes, the St. Lawrence lowlands, Newfoundland and Labrador. The zoogeography of North American freshwater fishes.

[b59] Van Oosterhout C, Hutchinson WF, Wills DPM, Shipley P (2004). MICRO-CHECKER: software for identifying and correcting genotyping errors in microsatellite data. Mol. Ecol. Notes.

[b60] Walter RP, Cena CJ, Morgan GE, Heath DD (2012). Historical and anthropogenic factors affecting the population genetic structure of Ontario's inland lake populations of walleye (*Sander vitreus*. J. Hered.

[b61] Ward RD, Woodwark M, Skibinski DOF (1994). A comparison of genetic diversity levels in marine, freshwater, and anadromous fishes. J. Fish Biol.

[b62] Watanabe K, Monaghan MT, Takemon Y, Omura T (2010). Dispersal ability determines the genetic effects of habitat fragmentation in three species of aquatic insect. Aquat. Conserv.

[b63] Weir BS, Cockerham CC (1984). Estimating *F*-statistics for the analysis of population structure. Evolution.

[b64] Wiens JA, Hanski I, Gilpin ME (1997). Metapopulation dynamics and landscape ecology. Metapopulation biology: ecology, genetics, and evolution.

[b65] Witt JDS, Zemlak RJ, Taylor EB (2011). Phylogeography and the origins of range disjunctions in a north temperate fish, the pygmy whitefish (*Prosopium coulterii*), inferred from mitochondrial and nuclear DNA sequence analysis. J. Biogeogr.

[b66] Zamudio KR, Robertson JM, Chan LM, Sazima I (2009). Population structure in the catfish *Trichogenes longipinnis*: drift offset by asymmetrical migration in a tiny geographic range. Biol. J. Linn. Soc.

