# Supplementary material for: Hierarchical analysis of genetic structure in the habitat-specialist Eastern Sand Darter (Ammocrypta pellucida)
Source: Ecol Evol. 2015 Jan 13;5(3):695–708. doi: 10.1002/ece3.1392 (PMC4328772; doi:10.1002/ece3.1392)
Supplement: Supplementary file 2 [file ece30005-0695-sd2.doc]

Supplementary table 1: Characterization of ten microsatellite markers used for genetic analysis of *Ammocrypta pellucida*. GeneBank Accession numbers, primer sequences, repeat motif, optimal magnesium chloride concentrations, and annealing temperatures for each locus was determined. Allele frequency range and number of populations significantly deviating from HWE equilibrium following Bonferroni correction were calculated for each microsatellite.

| Locus | GenBank Accession | Primer sequence (5'-3') | Repeat motif | MgCl_2_ | Ta(^o^C) | Allele Range (bp) | No. of alleles | HWE dev |
| --- | --- | --- | --- | --- | --- | --- | --- | --- |
| EosC6 | EF570435 | F: AAAGCCTGAGGGACAATTACAC | (CATC)_13_ | 2.2 | 58.0 | 265-349 | 12 | 0/40 |
|  |  | R: CCTTTGCTGGTAAATCTCACAC |  |  |  |  |  |  |
| EosC112 | EF570437 | F: CATGCAGGTATGCACACGTA | (AC)_4..._(GGTA)_11_ | 2.2 | 58.0 | 165-181 | 8 | 3/40 |
|  |  | R: GGCAGTGGTGAGACAGAAAC |  |  |  |  |  |  |
| EosD107 | EF570444 | F: CATTTAACATTCCCTGGTTGTG | (TAGA)_14_ | 2.2 | 53.0 | 251-323 | 18 | 0/40 |
|  |  | R: TTGCAGTGCAGTGGAGTTTTA |  |  |  |  |  |  |
| Esc132b | EF421255 | F: GAAGCACCTCACCAAACAGCG | (CTAT)_33_ | 2.2 | 56.6 | 144-212 | 18 | 0/40 |
|  |  | R: CCACACTGACACTGTGGACTGAC |  |  |  |  |  |  |
| Esd3 | HM775312 | F: CAGCTGAGGTGTATACAAAACAAT | (TC)_17_ | 2.1 | 59.5 | 172-214 | 13 | 0/40 |
|  |  | R: CAAAGCCTGCATGACAAAAA |  |  |  |  |  |  |
| Esd17 | HM775313 | F: ACCCCCATCGGACTAATGTT | (CA)_12_ | 2.1 | 58.2 | 142-342 | 70 | 1/40 |
|  |  | R: ATGTGTTGGTCCCTGAAAGC |  |  |  |  |  |  |
| Esd18 | HM775314 | F: CCTGATGATTGAGATTGATGATG | (GATA)_9_(AC)_12_ | 2.1 | 55.0 | 173-251 | 37 | 0/40 |
|  |  | R: GAAGCACGCACATTCAGAAA |  |  |  |  |  |  |
| Esd25 | HM775315 | F: TCATTCCACACCGTAACACG | (CA)_20_ | 2.1 | 58.9 | 72-110 | 20 | 0/40 |
|  |  | R: TAGGACTGCCAGGTTGTGC |  |  |  |  |  |  |
| Esd13 | JQ439945 | F: GTGGCTCCAAGATGCAAAGT | (GT)_15_ | 2.1 | 61.0 | 127-163 | 9 | 2/40 |
|  |  | R: CCGCTCAGGGATCTAGTCTG |  |  |  |  |  |  |
| EosD11 | EF570443 | F: ACCAGATGCAGTGGATGAATAT | (TAGA)_18_ | 2.2 | 53.0 | 206-314 | 22 | 2/40 |
|  |  | R: GCGGTATCTAATGCTATTTCCC |  |  |  |  |  |  |
